# Usefulness of P‐wave duration in patients with sick sinus syndrome as a predictor of atrial fibrillation

**DOI:** 10.1002/joa3.12604

**Published:** 2021-07-21

**Authors:** Yosuke Murase, Hajime Imai, Yasuhiro Ogawa, Naoaki Kano, Keita Mamiya, Tomoyo Ikeda, Kei Okabe, Kenji Arai, Shinji Yamazoe, Jun Torii, Katsuhiro Kawaguchi

**Affiliations:** ^1^ Department of Cardiology Komaki City Hospital Komaki Japan

**Keywords:** atrial fibrillation, electrocardiogram, pacemaker, P‐wave duration, sick sinus syndrome

## Abstract

**Background:**

This study aimed to clarify P‐wave duration (PWD) ability before pacemaker implantation to predict worsening atrial fibrillation (AF) burden after the procedure.

**Methods:**

We retrospectively investigated 75 patients who underwent permanent pacemaker implantation due to sick sinus syndrome (SSS) at Komaki City Hospital between January 2006 and May 2019. Worsening AF burden was defined as an increase in the number of AF episodes, each lasting ≥5.5 hours a day.

**Results:**

In the study population, 17 patients (23%) had worsening AF burden during the follow‐up period. These patients had significantly longer PWD in lead Ⅱ (117.9 ± 19.9 ms vs 101.3 ± 20.0 ms, *P* = .002) than the patients without worsening AF burden. The best discriminative cutoff value for PWD in lead Ⅱ was 108 ms (sensitivity, 77%; specificity, 67%). In multivariate analysis, PWD in lead II ≥108 ms (hazard ratio, 5.395; 95% confidence interval, 1.352‐21.523; *P* = .017) was an independent predictor of worsening AF burden. Patients with PWD in lead II <108 ms showed a significantly higher event‐free rate against worsening AF burden than those with PWD in lead II ≥108 ms (81% vs 9%, *P* = .005).

**Conclusions:**

Prolonged PWD before pacemaker implantation was the most important independent predictor of worsening AF burden after the procedure. In patients with SSS, prolonged PWD can be a useful marker for predicting worsening of AF burden after pacemaker implantation.

## 1 INTRODUCTION

Atrial fibrillation (AF) is the most commonly encountered arrhythmia. The number of AF patients increases among the higher age groups, and it is projected to reach 1 million people in 2050.^1^ AF is associated with increased mortality, heart failure, stroke, and decreased quality of life.^2^, ^3^ AF is frequently observed in patients with permanent pacemakers, and it carries the risk of heart failure hospitalization and stroke.^4^, ^5^ Managing AF is equally essential in patients with pacemakers and those without pacemakers. In patients with pacemakers, a higher percentage of ventricular pacing increased the risk of AF occurrence.^6^ Therefore, we attempted to program AV delay prolongation to avoid the high percentage of ventricular pacing in patients maintained by atrioventricular conduction. However, AF episodes were occasionally detected in patients with a lower percentage of ventricular pacing. Thus, the risk factors of AF occurrence are still unclear in patients with pacemakers.

Atrial structural and electrical remodeling are essential factors in the pathogenesis of AF. Atrial remodeling progression causes atrial conduction heterogeneity,^7^ which manifests as changes in the P‐wave morphology on electrocardiogram (ECG). P‐wave duration (PWD) has been demonstrated to be a reliable and noninvasive marker for predicting the incidence of AF.^8^ Kaypakli et al^9^ reported that prolonged PWD was associated with AF recurrence after cryoballoon ablation. This study aimed to clarify the predictive ability of PWD before pacemaker implantation on worsening AF burden after the procedure.

## 2 METHODS

### 2.1 Patient population

We retrospectively investigated 75 patients who underwent permanent pacemaker implantation due to sick sinus syndrome (SSS) at Komaki City Hospital between January 2006 and May 2019. Pacemaker implantation was performed for patients with symptomatic SSS, such as sinus bradycardia, sinoatrial block, sinus arrest, or bradycardia‐tachycardia syndrome. Exclusion criteria were as follows: (1) second or third‐degree atrioventricular block, (2) persistent or permanent AF, (3) junctional rhythm, (4) history of ventricular tachycardia, (5) intake of antiarrhythmic drugs before pacemaker implantation, (6) previous catheter ablation or prior heart surgery, (7) severe valvular heart disease, and (8) left ventricular ejection fraction <35%. Informed consent was obtained from all patients before the procedure, in accordance with our institutional guidelines. This study was performed in accordance with the Declaration of Helsinki.

### 2.2 Electrocardiographic assessment

**FIGURE 1:**
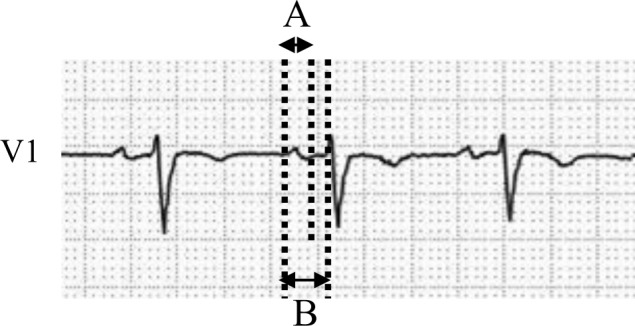
Schematic representation of P‐wave duration in lead V1. A is the P‐wave duration, and B is the PR interval

Twelve standard surface ECG leads were recorded before the procedure in all patients. Patients who received antiarrhythmic drugs before pacemaker implantation were excluded. Therefore, all ECGs were recorded without the influence of antiarrhythmic drugs. The ECG was digitally recorded with a paper speed and scale at 25 mm/s and 10 mm/mV, respectively (ECG‐2550; Nihon Kohden). The PR interval, PWD, and P‐wave amplitude were measured manually using a digital caliper in leads V1 and Ⅱ. The P‐wave duration index (PWDI) was calculated by dividing the PWD by the PR interval. The P‐wave was between the initial upward or downward point from the isoelectric line and the returning point to the isoelectric line. The isoelectric line was defined as the beginning of the P‐QRS complex to the end of the T‐wave (Figure 1).

### 2.3 Procedure of pacemaker implantation

The indication for pacemaker implantation was symptomatic SSS. The pacemaker devices used were manufactured by Medtronic, Inc, St. Jude Medical, Inc, or Abbott, Inc The right atrial lead was placed in the right atrial appendage, and the right ventricular lead was placed in the low‐septum or apex. Devices were programmed with pacing mode DDD and prolonged atrioventricular delay, managed by ventricular pacing (MVP^TM^, Medtronic) or ventricular intrinsic preference (VIP^TM^, St. Jude Medical or Abbott) mode to minimize ventricular pacing.

### 2.4 Patient follow‐up

The patients were hospitalized under continuous rhythm monitoring for 4 days after the procedure. After hospital discharge, all patients were scheduled for follow‐up visits. Device interrogations were performed 1, 6, and 12 months after pacemaker implantation, and then every 6 months. During device interrogations, atrial/ventricle lead parameters, percentages of atrial and ventricular pacing, automatic mode switch episodes, the burden of AF episodes, and noise episodes were recorded. If patients noticed any rhythm disorders in between follow‐up visits, they were recommended to arrange an early visit to the hospital for device interrogation. Worsening AF burden was defined as an increase in the number of AF episodes, with each episode lasting ≥5.5 hours a day.^10^ The increase in the number of AF episodes was the comparison of the number of AF episodes in the first follow‐up visit after pacemaker implantation and in the last follow‐up visit. Patients received antiarrhythmic drugs, catheter ablation, or anti‐tachycardia pacing (ATP), if necessary.

### 2.5 Statistical analysis

Continuous variables are presented as mean ± standard deviation. Categorical variables are presented as percentages. A chi‐square test was performed to compare categorical variables, and a Mann‐Whitney *U* test was performed to compare continuous variables. In this study, we used the receiver‐operating characteristic (ROC) curve analysis to determine the cutoff value. This method calculates the distance between the point (0, 1) and the point of cutoff value defined as the point on ROC curve where the distance is at a minimum. The factors shown to have a *P*‐value of <.05 in the univariate analysis were further assessed using multivariate analysis. The event‐free survival rate was estimated using the Kaplan‐Meier method and compared to the recurrence rate using a log‐rank test. Statistical analyses were performed using SPSS version 25 (SPSS Inc). A *P*‐value of <.05 was considered statistically significant.

## 3 RESULTS

### 3.1 Patient characteristics

A comparison of the baseline demographic and clinical characteristics between the patients with and without worsening AF burden is presented in Table 1. In the study population, 17 patients (23%) had worsening AF burden during the follow‐up period. The patients with worsening AF burden had a significantly higher age than the patients without AF burden (79 ± 6 years vs 74 ± 11 years, *P* = .016). They also had a higher proportion of hypertension patients (59% vs 31%, *P* = .037) and history of AF (88% vs 53%, *P* = .01). In addition, AF burdens in the first follow‐up visit after pacemaker implantation showed significantly higher in the patients with worsening AF burden (23.0 ± 36.9% vs 5.0 ± 12.2%, *P* < .001). In terms of echocardiographic parameters, the left ventricular ejection fraction (LVEF) was significantly lower in the patients with worsening AF burden (61.0 ± 11.6% vs 68.3 ± 7.3%, *P* = .021). The left atrial diameter and left atrial volume index were similar between the two groups. In addition, the number of patients prescribed with β‐blockers after pacemaker implantation was significantly higher in the patients with worsening AF burden (71% vs 29%, *P* = .002). The other clinical and echocardiographic parameters, and the details of antiarrhythmic therapy, such as antiarrhythmic drugs, catheter ablation, and ATP, were similar between the two groups.

**TABLE 1:** Comparison of baseline demographic and clinical characteristics between the patients with and without worsen AT/AF burden in the study population

Parameters	All patients, *n =* *75*	Worsen AT/AF burden, *n =* *17*	Without worsen AT/AF burden, *n =* *58*	*P* value
Age, years	75 ± 11	79 ± 6	74 ± 11	.016
Male	45 (60%)	10 (59%)	35 (60%)	.91
Hypertension	28 (37%)	10 (59%)	18 (31%)	.037
Congestive heart failure	11 (15%)	4 (24%)	7 (12%)	.24
Diabetes mellitus	15 (20%)	6 (35%)	9 (16%)	.073
Chronic kidney disease	8 (11%)	3 (18%)	5 (9%)	.289
Stroke/TIA	7 (9%)	1 (6%)	6 (10%)	.578
CHADS_2_ score	1.6 ± 1.1	1.9 ± 1.1	1.5 ± 1.0	.091
CHA_2_DS_2_‐VASc score	2.8 ± 1.4	3.2 ± 1.4	2.7 ± 1.0	.133
History of AF	46 (61%)	15 (88%)	31 (53%)	.01
BNP, pg/mL	93.1 ± 111.9	90.5 ± 46.4	93.5 ± 120.2	.481
eGFR, mL/min/1.73 m^2^	57.3 ± 19.6	51.3 ± 13.5	58.5 ± 20.5	.137
Hb, g/dL	13.1 ± 1.8	13.5 ± 1.8	13.0 ± 1.8	.317
Echocardiographic parameters				
LVEF, %	66.5 ± 9.1	61.0 ± 11.6	68.3 ± 7.3	.021
LAD, mm	38.5 ± 5.4	38.8 ± 5.9	38.4 ± 5.3	.633
LA volume index, mL/m^2^	37.0 ± 12.5	34.0 ± 9.1	37.7 ± 13.2	.484
E/e′	14.6 ± 9.4	14.1 ± 6.8	14.7 ± 10.0	.89
Antiarrhythmic therapy after device implantation				
β‐blocker	29 (39%)	12 (71%)	17 (29%)	.002
Antiarrhythmic drug	20 (27%)	5 (29%)	15 (26%)	.801
Class Ⅰ	8 (11%)	0 (0%)	8 (14%)	.105
Class Ⅲ	12 (16%)	5 (29%)	7 (12%)	.086
Catheter ablation	21 (28%)	3 (18%)	18 (31%)	.28
ATP	18 (24%)	7 (41%)	11 (19%)	.059
Parameters after device implantation				
Atrial pacing ratio in first follow‐up visit, %	60 ± 31	60 ± 30	60 ± 31	.994
AF burdens in first follow‐up visit, %	9.0 ± 21.4	23.0 ± 36.9	5.0 ± 12.2	<.001
AF burdens in last follow‐up visit, %	13.5 ± 30.0	50.5 ± 45.8	2.6 ± 7.0	<.001

Note

Values are mean ± SD or number (percentage).

Abbreviations: AF, atrial fibrillation;AT, atrial tachycardia; ATP, antitachycardia pacing; BNP, brain natriuretic peptide; eGFR, estimated glomerular filtration rate; Hb, Hemoglobin; LA, left atrium; LAD, left atrial diameter; LVEF, left ventricular ejection fraction; TIA, transient ischemic attack.

### 3.2 ECG parameters before pacemaker implantation

**FIGURE 2:**
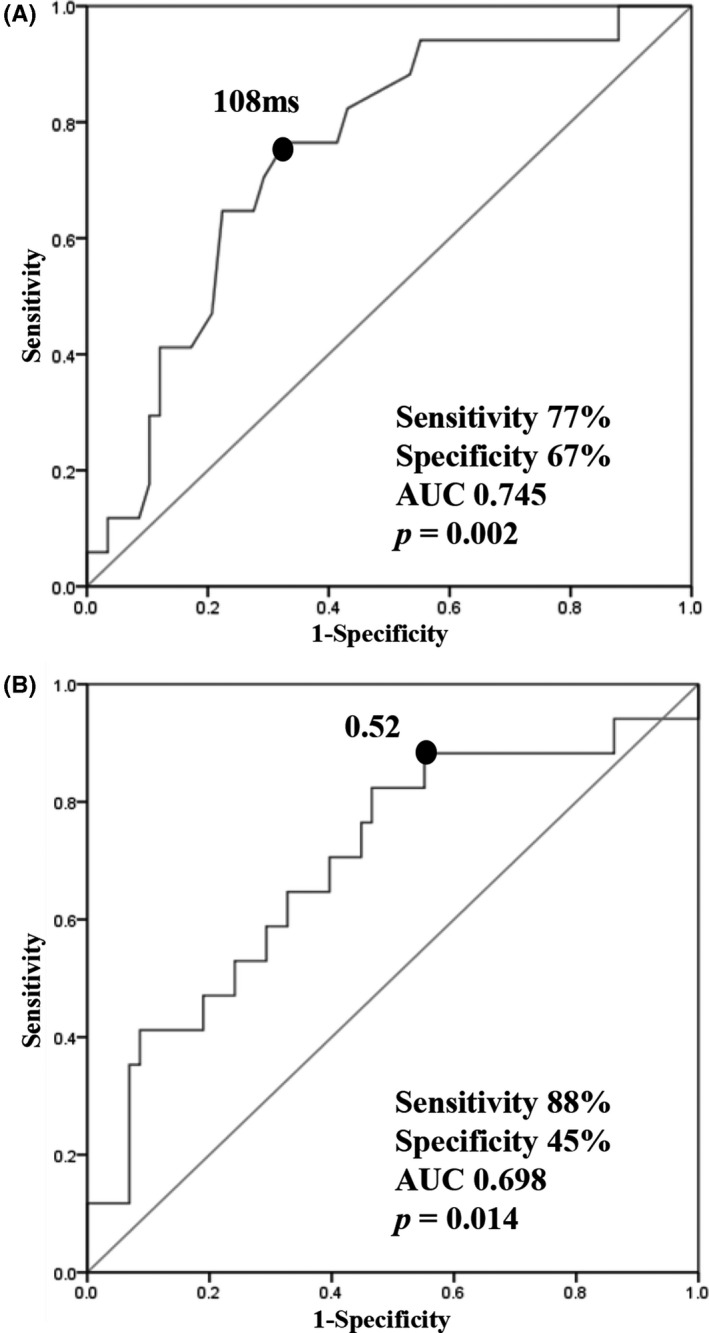
(A) ROC curve of PWD in lead Ⅱ for worsening AF burden after pacemaker implantation. (B) ROC curve of PWDI in lead Ⅱ for worsening AF burden after pacemaker implantation. AF, atrial fibrillation; AUC, area under the curve; PWD, P‐wave duration; PWDI, P‐wave duration index; ROC, receiver‐operating characteristic

A comparison of the ECG parameters of the P‐wave indices is shown in Table 2. The PR interval, number of patients with first‐degree atrioventricular block, P‐wave amplitude in leads V1 and Ⅱ, PWD, and PWDI in lead V1 were similar between the two groups. In patients with worsening AF burden, the PWD in lead Ⅱ was significantly longer (117.9 ± 19.9 ms vs 101.3 ± 20.0 ms, *P* = .002), and the PWDI in lead Ⅱ was significantly larger (0.65 ± 0.14 vs 0.56 ± 0.12, *P* = .014). ROC curve analysis was performed to evaluate the correlation between PWD in lead Ⅱ and worsening AF burden after pacemaker implantation. We set the cutoff values of PWD and PWDI in lead Ⅱ to 108 ms (sensitivity, 77%; specificity, 67%; Figure 2A) and 0.52 (sensitivity, 88%; specificity, 45%; Figure 2B), respectively.

**TABLE 2:** Comparison of electrocardiographic parameters about P‐wave indices

Parameters	All patients *n =* *75*	Worsen AT/AF burden *n =* *17*	Without worsen AT/AF burden *n =* *58*	*P* value
PR interval, ms	185.6 ± 43.3	188.9 ± 42.7	184.6 ± 43.8	.709
First‐degree atrioventricular block	21 (28%)	5 (29%)	16 (28%)	.883
P‐wave amplitude in V1, mV	0.12 ± 0.06	0.11 ± 0.05	0.13 ± 0.06	.305
P‐wave amplitude in Ⅱ, mV	0.13 ± 0.05	0.12 ± 0.05	0.13 ± 0.05	.142
PWD in V1, ms	102.9 ± 23.4	106.6 ± 24.6	101.8 ± 23.2	.326
PWD in Ⅱ, ms	105.1 ± 21.0	117.9 ± 19.9	101.3 ± 20.0	.002
PWDI in V1	0.57 ± 0.13	0.58 ± 0.14	0.57 ± 0.13	.631
PWDI in Ⅱ	0.58 ± 0.13	0.65 ± 0.14	0.56 ± 0.12	.014

Note

Values are mean ± SD or number (percentage).

Abbreviations: AF, atrial fibrillation;#AT, atrial tachycardia; PWD, p‐wave duration; PWDI,#p‐wave duration index.

### 3.3 The predictors of worsening AF burden after pacemaker implantation

Univariate and multivariate Cox regression analyses revealed the predictors of worsening AF burden after pacemaker implantation. Univariate analysis showed that hypertension (hazard ratio [HR], 3.175; 95% confidence interval CI, 1.041‐9.677; *P* = .042, history of AF (HR, 6.532; 95% CI, 1.369‐31.180; *P* = .019), PWD in lead II ≥108 ms (HR, 6.671; 95% CI, 1.916‐23.229; *P* = .003), and AF burdens in first follow‐up visit (HR, 1.034; 95% CI, 1.006‐1.062; *P* = .017) were significantly associated with worsening AF burden (Table 3). PWDI was excluded from this analysis to eliminate confounding factors. In multivariate analysis, PWD in lead II ≥108 ms (HR, 5.395; 95% CI, 1.352‐21.523; *P* = .017) was an independent predictor of worsening AF burden (Table 4).

**TABLE 3:** Univariate Cox regression analyses for worsening AT/AF burden after pacemaker implantation

Parameters	Univariate analysis	
	HR (95% CI)	*P* value
Age	1.11 (0.994‐1.239)	.063
Male	1.065 (0.355‐3.200)	.91
Hypertension	3.175 (1.041‐9.677)	.042
Congestive heart failure	2.242 (0.569‐8.832)	.249
Diabetes mellitus	2.97 (0.874‐10.085)	.081
Chronic kidney disease	2.271 (0.483‐10.678)	.299
Stroke/TIA	0.542 (0.061‐4.839)	.583
History of AT/AF	6.532 (1.369‐31.180)	.019
BNP	1 (0.991‐1.009)	.956
eGFR	0.982 (0.945‐1.020)	.344
LVEF	0.91 (0.845‐0.979)	.011
LAD	1.013 (0.912‐1.125)	.809
LA volume index	0.987 (0.948‐1.026)	.501
E/e'	0.972 (0.911‐1.038)	.398
PR interval	1.002 (0.991‐1.012)	.773
First‐degree atrioventricular block	1.173 (0.411‐3.348)	.766
PWD in Ⅱ ≥108 ms	6.671 (1.916‐23.229)	.003
Atrial pacing ratio in first follow‐up visit	1.000 (0.981‐1.020)	.969
AF burdens in first follow‐up visit	1.034 (1.006‐1.062)	.017
Antiarrhythmic drug	1.390 (0.710‐2.720)	.337
ATP	2.991 (0.930‐9.616)	0.066

Abbreviations: AF, atrial fibrillation; AT, atrial tachycardia; ATP, antitachycardia pacing; BNP, brain natriuretic peptide; eGFR, estimated glomerular filtration rate; LA, left atrium; LAD, left atrial diameter; LVEF, left ventricular ejection fraction; PWD P‐wave duration; TIA transient ischemic attack.

**TABLE 4:** Multivariate Cox regression analyses for worsening AT/AF burden after pacemaker implantation

Parameters	Multivariate analysis	
	HR (95% CI)	*P* value
Hypertension	2.268 (0.510‐10.089)	.282
History of AT/AF	8.974 (0.940‐85.702)	.057
LVEF	0.972 (0.892‐1.059)	.518
PWD in Ⅱ ≥108 ms	6.528 (1.400‐30.429)	.017
AF burdens in first follow‐up visit	1.020 (0.988‐1.053)	.232

Abbreviations: AF, atrial fibrillation; AT, atrial tachycardia; LVEF, left ventricular ejection fraction; PWD, P‐wave duration.

### 3.4 The association of PWD with worsening AF burden after pacemaker implantation

**FIGURE 3:**
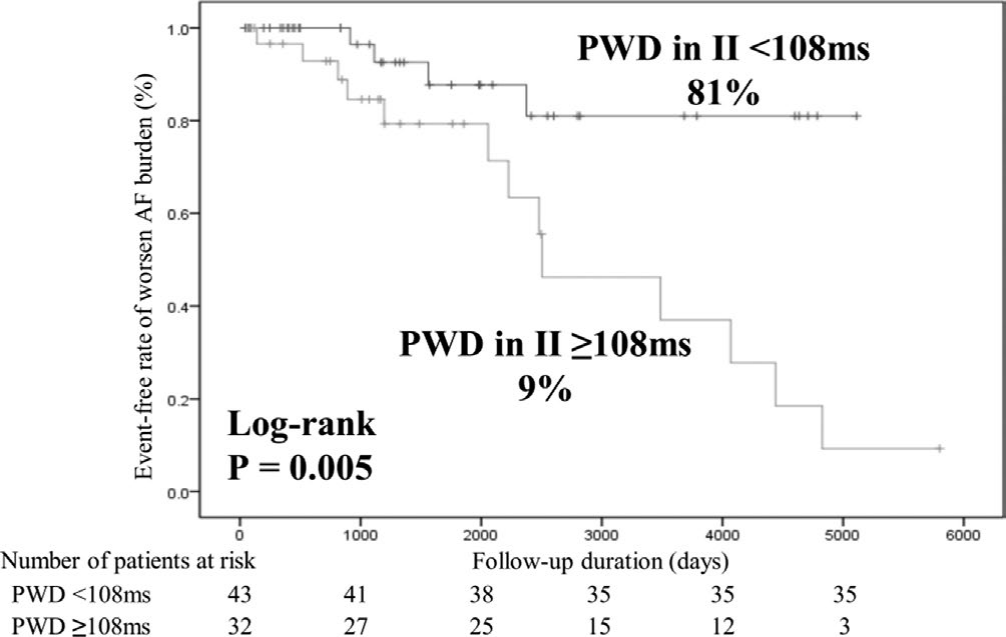
Kaplan‐Meier curves of the survival‐free rate of worsening AF burden after pacemaker implantation between the two groups (PWD in lead Ⅱ <108 ms; PWD in lead Ⅱ ≥108 ms). AF, atrial fibrillation; PWD, P‐wave duration

A Kaplan‐Meier analysis was performed to evaluate the event‐free rate of patients with worsening AF burden after pacemaker implantation. Patients with PWD in lead Ⅱ <108 ms exhibited a significantly higher event‐free rate than those with PWD in lead II ≥108 ms (81% vs 9%; *P* = .005; Figure 3).

## 4 DISCUSSION

This study aimed to demonstrate the relationship between PWD and worsening AF burden in patients with SSS. The study found that prolonged PWD before pacemaker implantation was the most important independent predictor of worsening AF burden after the procedure.

ECG can be obtained noninvasively. Previous reports have shown that P‐wave indices such as the PR interval,^11^ P‐wave axis,^12^ and P‐wave terminal force in V1^13^ are related to AF. The PWD is a noninvasive marker of AF recurrence after catheter ablation.^9^ Demirtas et al^14^ reported that a prolonged PWD was associated with the incidence of silent AF episodes in patients with cardiac resynchronization therapy defibrillators. Several studies have reported a relationship between the PWD and worsening AF burden in patients with SSS. Kristensen et al^15^ and Padeletti et al^16^ reported that a prolonged PWD was predictor of AF after pacemaker implantation in patients with SSS. The results of our study are consistent with those of previous reports. However, the definition of AF in the current study differed from that in other studies. Kristensen et al^15^ defined AF as an atrial high rate and mode switching episode. Padeletti et al^16^ defined the outcome as AF‐related hospitalization and cardioversion. However, our study defined a worsening AF burden as an increase in the number of AF episodes, with each episode lasting ≥5.5 hour per day. This definition of worsening AF burden was the same as that in the TRENDS study.^10^ Thus, a prolonged PWD was a predictor of worsening AF burden and such patients may be at greater risk of thromboembolic events in the future. This conclusion differed from those of previous reports.

SSS patients with frequent AF episodes have an increased risk of worsening symptoms, heart failure, and stroke. Moreover, they receive antiarrhythmic drugs, pacemaker implantation, and catheter ablation as needed. In patients with pacemaker implantation, right ventricular pacing >40% was a risk factor for AF.^17^ Therefore, patients with SSS are programmed to minimize ventricular pacing after pacemaker implantation. In addition, right atrial septum pacing was associated with a lower risk of AF in SSS compared to right atrial appendage pacing.^18^ However, all patients in this study had a low percentage of ventricular pacing, and the atrial lead was placed at the right atrial appendage. Thus, the patient characteristics in terms of pacemaker operation and management did not significantly differ in this study population. We revealed that the PWD in lead Ⅱ was an independent predictor of worsening AF burden in patients with SSS. Based on this study's findings, we clarified the risk stratification of worsening AF burden before pacemaker implantation. This comes with the benefit of administering antiarrhythmic and anticoagulant therapy after the procedure. In this study, the percentage of patients who received antiarrhythmic drugs, catheter ablation, or programmed ATP was <30%. Aggressive antiarrhythmic and anticoagulant therapy can be administered in patients who are likely to develop worsening AF burden before pacemaker implantation.

P‐waves represent electrical conduction from the sinus node to the atrioventricular node and characterizes atrial depolarization. Moreover, PWD reflects intra‐atrial conduction time. Jadidi A et al^19^ reported that prolonged PWD was significantly associated with intra‐atrial conduction delay and advanced low‐voltage substrate of the left atrium. Therefore, prolonged PWD was related to electrical and structural remodeling. Electrical and structural remodeling of the atrium is a consequence of sustained AF. Atrial electrical remodeling is characterized by shortening of the atrial refractory period^20^ and fibrosis development,^21^ which are essential factors for initiating and maintaining AF. In patients with progressive atrial remodeling, the sinus rhythm was difficult to restore with antiarrhythmic drugs, electrical cardioversion, or catheter ablation. In this study, patients with prolonged PWD exhibited worsening AF burden during the follow‐up period. Patients with advanced intra‐atrial conduction delay tended to have a worsened AF burden despite antiarrhythmic therapy. This result suggested that the pathogenesis of prolonged PWD involved the progression of atrial remodeling. However, the patients in this study did not exhibit significant differences in echocardiographic parameters. Echocardiographic parameters, such as left atrial diameter and left atrial volume, reflect structural remodeling. One hypothesis is that the mechanism of electrical remodeling is separate from that of structural remodeling. The progression from paroxysmal to persistent AF is associated with progressive atrial remodeling, which leads to higher fibrillatory wave frequencies^22^ and enlargement of the left atrium size. Previous reports showed that atrial electrical remodeling developed quickly,^23^ but structural remodeling, resulting in the left atrium's enlargement, was sustained over a long period.^24^ In other words, the progression of atrial electrical remodeling occurs prior to extended structural remodeling. This study revealed that prolonged PWD was the most important predictor of worsening AF burden. This was consistent with previous studies. In patients with SSS, the PWD in lead Ⅱ was a useful marker for predicting the worsening of AF burden after pacemaker implantation.

This study had some limitations. First, this was a retrospective and single‐center study. The sample size was relatively small due to the study design and strict exclusion criteria. Additionally, patients whose PWD were not measured, such as patients with junctional rhythm and AF, were excluded from this study. Thus, the number of patients included was limited. However, we eliminated the influence of patient characteristics, such as the atrial lead position and percentage of atrial or ventricular pacing. Second, the measurement of P‐wave indices was performed manually. This limitation potentially affected the relationship between the value of PWD and the incidence rate of worsening AF burden. This possibly caused the low AUC values in the ROC curve analysis. Third, surface ECG in leads V1 and II were analyzed, but other surface ECG leads were not assessed in this study. However, previous studies that investigated the relationship between PWD and the occurrence of AF used lead Ⅱ.^9^, ^14^ The findings of this study were consistent with those of previous studies. Finally, we assessed atrial electrical remodeling from PWD in lead Ⅱ. However, we did not sufficiently evaluate structural remodeling effects, such as scars or the low‐voltage area in the left atrium. Enhanced MRI or voltage mapping of the atrium is required to reveal the relationship between electrical and structural remodeling.

## 5 CONCLUSION

This study demonstrated the relationship between PWD in lead Ⅱ and worsening AF burden after pacemaker implantation in patients with SSS. Prolonged PWD before pacemaker implantation was the most important independent predictor of worsening AF burden after the procedure. In patients with SSS, prolonged PWD can be a useful marker for predicting the worsening of AF burden after pacemaker implantation.

## CONFLICT OF INTEREST

Authors declare no conflict of interests for this article.
